# A New Bioactive Complex between Zn(II) and a Fluorescent Symmetrical Benzanthrone Tripod for an Antibacterial Textile

**DOI:** 10.3390/ma12213473

**Published:** 2019-10-23

**Authors:** Desislava Staneva, Evgenia Vasileva-Tonkova, Ivo Grabchev

**Affiliations:** 1 Department of Chemical Technology, University of Chemical Technology and Metallurgy, 1756 Sofia, Bulgaria; grabcheva@mail.bg; 2 The Stephan Angeloff Institute of Microbiology, Bulgarian Academy of Sciences, 1113 Sofia, Bulgaria; evaston@yahoo.com; 3 Faculty of Medicine, Sofia University “St. Kliment Ohridski”, 1407 Sofia, Bulgaria

**Keywords:** benzanthrone, tripod, metal complex, antimicrobial activity, antibacterial textile, XPS, SEM

## Abstract

A new fluorescent Zn(II) complex of symmetrical tripod form based on a 3-substituted benzanthrone (BT) has been synthesized and characterised. The basic photophysical properties of the new metal complex have been determined. It has been found by fluorescence spectroscopy that, one zinc ion forms a complex with the tripod ligand. The surface morphology of the ligand and its Zn(II) complex has been investigated by the scanning electron microscopy (SEM) technique. X-ray photoelectron spectroscopy (XPS) has been used for the characterisation of the chemical composition of the complex surfaces. The antibacterial activity of the Zn(II) complex has been investigated in solution and upon its deposition onto a cotton fabric. A reduction of biofilm formation on the surface of the cotton fabric has been observed compared to the non-treated cotton material. The results obtained demonstrate that the studied Zn(II) complex possesses good antimicrobial activity being most effective against the used Gram-positive bacteria.

## 1. Introduction

Among the known fluorophore structures, the polarization of the chromophoric system of benzanthrone derivatives yields intense fluorescence emission combined with a bright colour [1,2] that, can vary widely. That determines their respective applications: in liquid-crystalline systems for electro-optical displays [3,4,5,6], as fluorescent solvatochromic probes for DNA intercalations [7], for membrane studies [8,9], as biomedical probes for proteins, lipids, and cells [10,11] or as sensors for aliphatic amines in an aqueous medium [12,13,14]. A second generation poly(propylene amine) dendrimer from modified with benzanthrone chromophore units has been investigated as a fluorescent dendrimer sensor for aliphatic amines and protons [15,16]. 

Over the last few decades, the steadily increasing resistance of a number of common pathogens to almost all commercial antimicrobials determine the need to look for new substances with more effective antimicrobial properties. The efforts of researchers are aimed at exploring the antimicrobial potential of different compounds so that new highly effective formulations could be duly developed. Different 3-aminosubstituted benzanthrone derivatives have been tested as antimicrobial agents against Gram-positive and Gram-negative bacteria and yeasts [15,17,18,19].

The antimicrobial properties of textile materials obtained via different treatment with biologically active compounds have been investigated and novel, more effective antimicrobial compounds have been obtained [20,21]. In this direction the use of textile fabrics as a support of benzanthrone compounds with antimicrobial activity opens the possibilities of preparing effective therapeutics for curing resistant infections. On the other hand, the use of such compounds to obtain colored antibacterial dressings and gauze would be a new challenge.

In this work, we describe the synthesis and characterisation of a new Zn(II) complex based on a symmetric ligand containing three benzanthrone units. Upon its deposition onto a cotton fabric the colour characteristics of the dyed fabric have been described. The antimicrobial activity of the zinc complex has also been evaluated in solution and after its deposition on a cotton fabric aiming to obtain an effective antibacterial textile. 

## 2. Experimental Part 

**Scheme 1:**
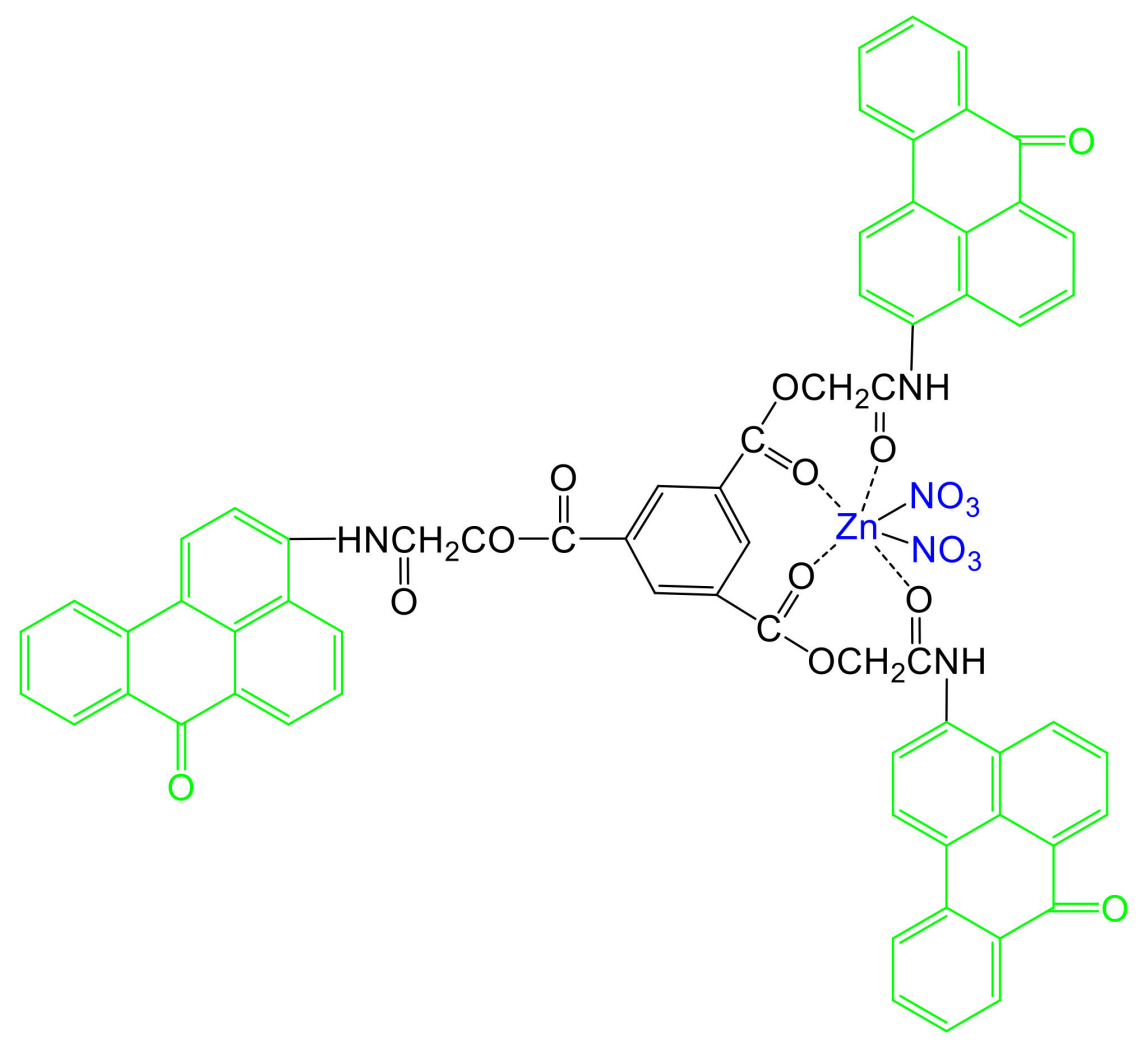
Chemical structure of [Zn(BT)(NO_3_)_2_].

The symmetrical tripod BT (Scheme 1) used in this study as a ligand for the synthesis of Zn(II) complex was synthesized according to literature procedures described recently [19]. 

### 2.1. Synthesis of Zn(II) Complex [Zn(BT)(NO_3_)_2_]

The complex was prepared by adding BT (1.06 g, 1 mmol) into Zn(NO_3_)_2_·5H_2_O. (0.31 g 1.1 mmol) 20 mL solution in ethanol. The mixture was stirred at 40 °C for 60 min. The precipitate was filtrated, washed three times with ethanol (10 mL) and then dried under vacuum at 40 °C. Yield: 96%. 

Fourier transform infrared (FTIR) spectroscopy cm^−1^: 3253, 3064, 1736, 1672, 1651, 1544, 1386, 1307, 1211, 974, 775, 732, 696.

^1^H nuclear magnetic resonance (NMR) (Dimethyl sulfoxide—DMSO-d6, ppm): 10.64 (br s, 3H, NH), 8.88 (bs, 3H, Ar), 8.80-8.60 (m, 9H, Ar), 8.53 (bs, 3H, Ar), 8.31 (bs, 3H, Ar), 8.07 (bs, 3H, Ar), 7.97 (bs, 3H, Ar), 7.85 (bs, 3H, Ar), 7.06 (bs, 3H, Ar), 5.27 (s, 6H, −CH_2_−), ^13^C NMR (DMSO-d6, ppm): 183.5, 167.2, 165.1, 155.3, 136.6, 135.2, 134.7, 132.5, 131.1, 130.8, 130.1, 128.6, 128.1, 128.0, 127.3, 126.2, 124.6, 124.2, 127.7, 65; API-ES-MS (Electrospray mass spectroscopic) (positive) m/z: found: 1255.813 ([M + H]^+^); 

Elemental analysis: C_66_H_39_N_5_O_18_Zn (1254.80 g mol^−1^): calculated (%): C 63.11, H 3.11, N 5.57; found (%): C 63.24, H 3.13, N 5.49.

### 2.2. Analysis

Ultraviolet–visible (UV–Vis) spectrophotometric investigations were performed on a “Thermo Spectronic Unicam UV 500” spectrophotometer. Emission spectra were taken on a “Cary Eclipse” spectrofluorometer. All spectra were recorded using 1 cm path length synthetic quartz glass cells at a 10^−5^ mol L^−1^ concentration. ^1^H NMR (600.13 MHz) and 13C (150.92 MHz) spectra were acquired on an AVANCE AV600 II + NMR spectrometer. The measurements were carried out in a DMSO-d6 solution at ambient temperature. The chemical shifts were referenced to a tetramethylsilane (TMS) standard. The X-ray photoelectron spectroscopy (XPS) measurements were performed in the vacuum chamber of an Escalab Mk II VG Scientific spectrometer, with an Mg anode (h = 1253 eV) as a radiation source. The pressure of the residual gases was 2 × 10^−7^ Pa. Electrospray mass spectroscopic (ES–MS) measurements were carried out using a Hewlett–Packard Series 110 0 MSD. The surface morphology of BT and its Zn(II) complex was analyzed on a scanning electron microscope (SEM) JSM-5510 (JEOL), operated at 10 kV of acceleration voltage. Before imaging, the investigated samples were coated with gold by JFC-1200 fine coater (JEOL). Zn(NO_3_)_2_·5H_2_O was used as sources for Zn(II) cations as obtained from Sigma-Aldrich. The effect of the Zn(II) ions upon the fluorescence intensity was examined by adding a few μL of stock solution (c = 10^−3^ mol/L) of the metal cations to a known volume of the ligand solution (3 mL). The addition was limited to 0.08 mL, so that dilution remained insignificant [22]. 

### 2.3. Purification of Cotton Fabric and Treatment with BT and [Zn(BT)(NO_3_)_2_]

The purification of cotton fabric (surface weight 140 g/m^2^) was carried out according to the described method [23]. Compound BT (0.005 g) or [Zn(BT)(NO_3_)_2_] was dissolved in 5 mL DMF (*N,N*-dimethylformamide). A cotton fabric sample (1 g) was immersed into the solution at 25 °C for 30 min, washed with water and dried at room temperature. The amount of deposited tripod on the cotton matrix was examined spectrophotometrically, by standard procedure of measuring the absorption of the dyestuff before and after treatment. It was found that 87% of the tripod initially used (0.0043 g) was deposited on the fabric.

### 2.4. Colour Measurements

A Datacolor Spectraflash SF300 spectrophotometer (Datacolor, NJ, USA) was used to determinate colour coordinates (L*a* b*, xy and XYZ) of the treated with BT and [Zn(BT)(NO_3_)_2_] cotton fabrics.

The intensity of the colour was expressed on the basis of Kubelka–Munk Equation (1) [24,25]:

K\S = (1 − R)^2^/2R (1)

where

K: is constant of the light absorption of the cotton fabric,
S: is constant of the light scattering of the cotton fabric,
R: is the reflectance of the cotton fabric, expressed in fractional form in the spectral range 400–700 nm.

The assessment of the coloured cotton fabrics was made in terms of tristimulus colorimetry. The colour range achieved was characterized quantitatively and qualitatively according to the three main parameters (L*, a* and b*) and the chromatisity coordinates (x and y). The colour coordinates L*, a*, b* were determined from the Equations (2)–(4):

L* = 116(Y/Y_0_)^1/3^ − 16 (2)

a* = 500[(X/X_0_)^1/3^ − (Y/Y_0_)^1/3^] (3)

b* = 200[(Y/Y_0_)^1/3^ − (Z/Z_0_)^1/3^] (4)

where Xo, Yo, Zo are the tristimulus values of specified achromatic light used in illumination, X, Y, Z are the values defined for the coloured polymers. The values of Yo is normalized in such a way that Yo = 100.

Color difference of the dyed cotton fabric is estimated by Equation (5) [26]:

ΔE∗ = [(ΔΛ∗)^2^ + (Δα∗)^2^ + (Δβ∗)^2^]^1/2^ (5)

where ΔL*, Δa*and Δb* are the difference in coordinates of dyed and non-dyed cotton fabric In the case of the non-dyed cotton fabric ΔE* = 0

### 2.5. Test Organisms

In this study, the following microbial strains were used in the antimicrobial assay (Collection of the Institute of Microbiology, Sofia, Bulgaria): Gram-positive bacteria *Bacillus subtilis* and *Bacillus cereus (B. cereus)*, Gram-negative bacteria *Pseudomonas aeruginosa (P. aeruginosa)* and *Escherichia coli (E. coli)*, and the yeasts *Candida lipolytica* and *Saccharomyces cerevisiae*.

### 2.6. Antimicrobial Activity Assay

The antimicrobial activity of the new [Zn(BT)(NO_3_)_2_] complex was quantitatively assessed by the minimum inhibitory concentrations (MIC_90_). Stock solution of the investigated complex (0.5% in DMSO) was prepared and different amounts were added into test tubes with sterile meat-peptone broth (MPB) to final concentrations of 100, 200, 280, 450 and 650 µg/mL. After inoculation, the tubes were incubated at appropriate temperature for 24 h. Then MIC_90_ was determined by measuring the turbidity at 600 nm (OD_600_) and confirmed by the cell counting method. Three independent experiments were conducted and an average was taken; standard deviations were less than 5%.

### 2.7. Antibacterial Activity Test of Textile Samples

The antibacterial effect of the obtained cotton sample impregnated in 0.5% solution of [Zn(BT)(NO_3_)_2_] was tested against *B. cereus*, *E. coli* and *P. aeruginosa* as model strains. The tests were conducted as described in a previous paper [16]. Antimicrobial tests were done in triplicate and the average was taken; standard deviations were less than 5%.

### 2.8. Preparation of Cotton Fabrics for Scanning Electron Microscopy (SEM)

Samples of untreated cotton fabric and cotton fabrics treated with BT and [Zn(BT)(NO_3_)_2_] were incubated for 24 h in MPB inoculated with *B. cereus* cell suspension. After incubation, the samples were washed, dried and coated with gold, and then investigated on a Jeol JSM-5510 scanning electron microscope (Jeol Ltd., Tokyo, Japan) at different magnification.

## 3. Results and Discussion 

### 3.1. Syntesis and Spectral Characterisations of [Zn(BT)(NO_3_)_2_]

The synthesis of a symmetrical tripod containing three benzanthrone units (BT) has been described recently [19]. This tripod has been used as a ligand for obtaining its Zn(II) complex [Zn(BT)(NO_3_)_2_] (Scheme 1). The synthesis of Zn(II) complex was conducted in an ethanol solution of BT ligand in the presence of zinc nitrate for 60 min at 40 °C. The solid precipitate was filtrated washed with ethanol and dried. The final product of [Zn(BT)(NO_3_)_2_] (Scheme 1) was analysed by FTIR, ^1^H NMR and ^13^C NMR spectroscopy absorption and fluorescence spectroscopy, XPS analysis, ES–MS and elemental analysis.

### 3.2. Spectral Analysis of [Zn(BT)(NO_3_)_2_]

**Figure 1:**
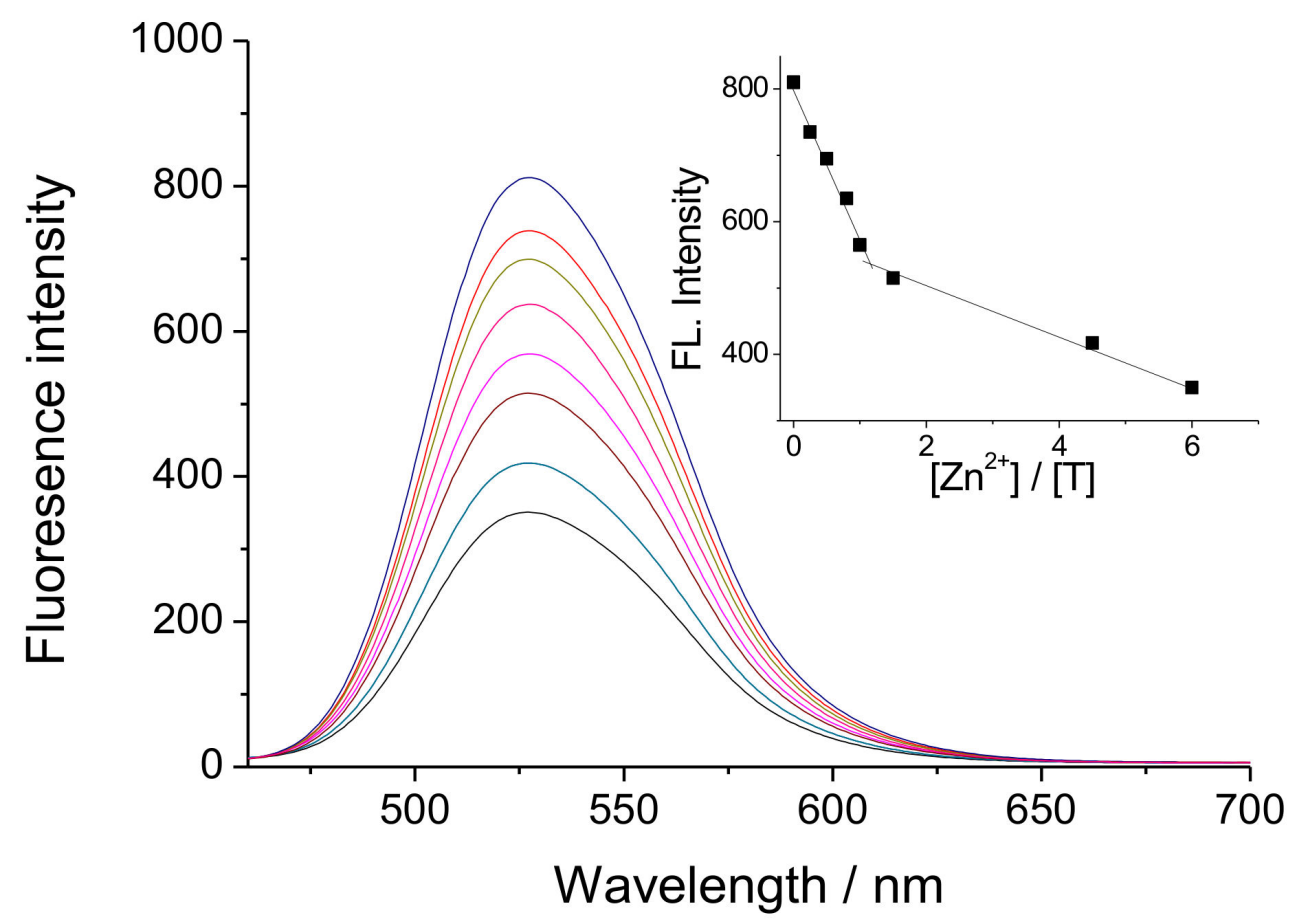
Fluorescence spectra of BT (c = 1 × 10^−5^ M) at different concentrations of Zn(II) ions (c = 0–6 × 10^−5^ M). The inset shows the quenching of the fluorescence intensity on Zn(II) concentrations.

DMF solution has been used as a solvent to evaluate the changes in the fluorescence intensity of BT in in the presence of different concentrations of Zn(II). As can be seen from Figure 1, a quenching of fluorescence intensity occurs upon titration of the solution with Zn(II) ions depending of its concentration. In this case, we assume that Zn(II) ions react with two amidic groups from BT ligand and form coordinative bonds with oxygen atoms from the carbonyl groups, thus forming a metal complex [Zn(BT)(NO_3_)_2_], as shown in Scheme 1. The inset of Figure 1 shows clearly that one Zn(II) ion forms a complex with BT ligand. The fluorescence-quenching in this case can be explained by the change in the electron density of the benzanthrone chromophor system, which leads to an increase in the electron acceptor nature of the amino substituent. Another reason is the possible conformation changes during complex formation. As seen from Scheme 1, one benzanthrone unit remains free in the complex and the total fluorescence for [Zn(BT)(NO_3_)_2_] is the sum of the fluorescence of complexing and free benzanthrone residues. The fluorescence maximum of [Zn(BT)(NO_3_)_2_] appears approximately at the same position as that of the BT ligand indicating that, the coordination of Zn(II) ions with the ligand does not change the chromophore system of benzanthrone molecules, but only affects the intensity of emitted fluorescence quenching more that 57% of it. The incomplete fluorescence quenching during the titration of ligand BT with Zn(II) ions can be explained by the fact that one benzanthrone fragment does not participate in the formation of coordinate bonds with the Zn(II) ions. 

### 3.3. X-ray Photoelectron Spectroscopy (XPS) Characterisation

**Figure 2:**
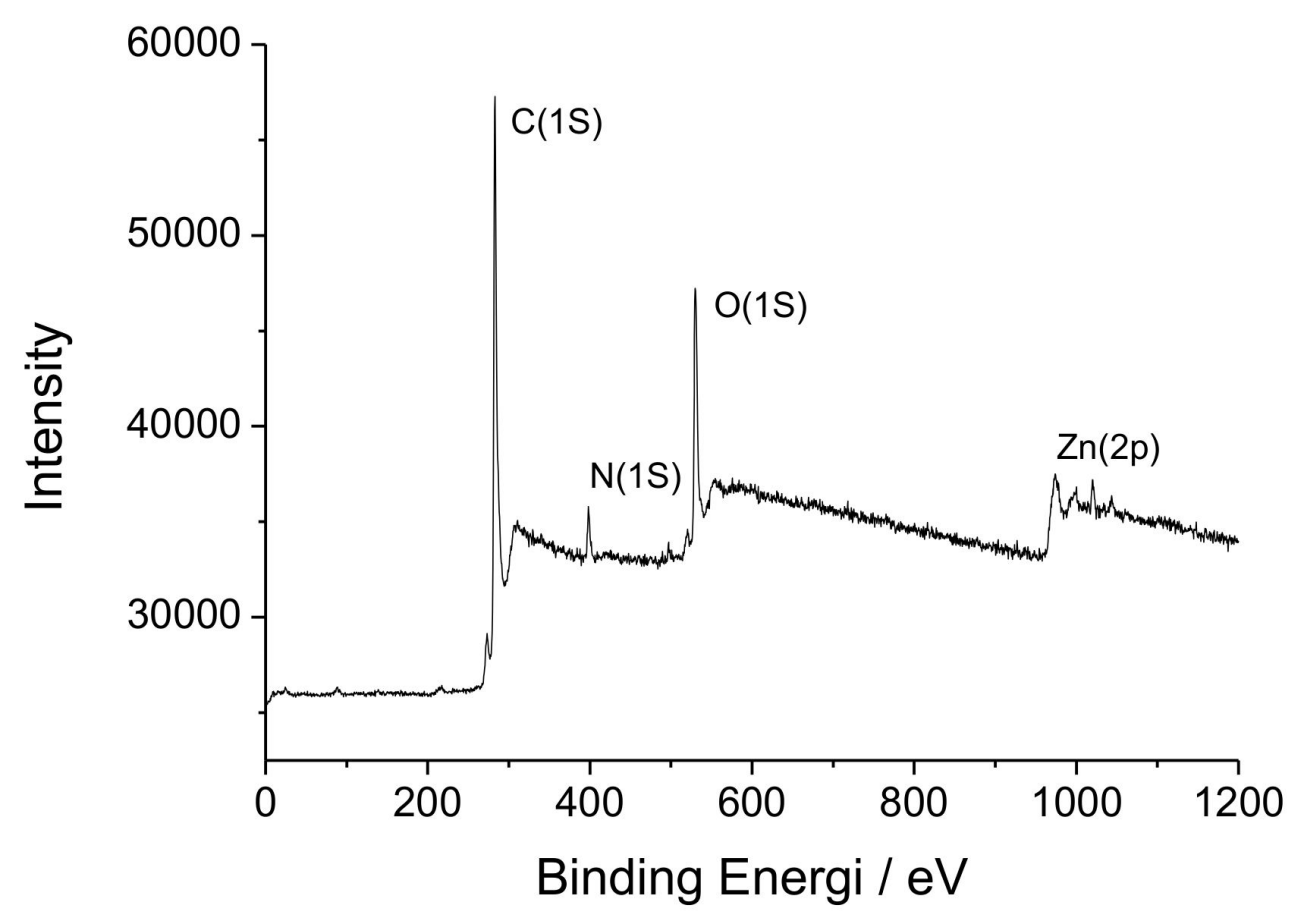
Overall X-ray photoelectron spectroscopy (XPS) spectrum of [Zn(BT)(NO_3_)_2_].

The chemical composition of surfaces of [Zn(BT)(NO_3_)_2_] has been investigated by XPS. Scheme 1 shows that, [Zn(BT)(NO_3_)_2_] contains only C, H, N, O, Zn elements. A wide scan XPS survey of [Zn(BT)(NO_3_)_2_] has been performed to confirm the presence of these elements. The respective characteristic peaks for Zn2p, O1s, N1s, and C1s are shown in Figure 2. 

**Figure 3:**
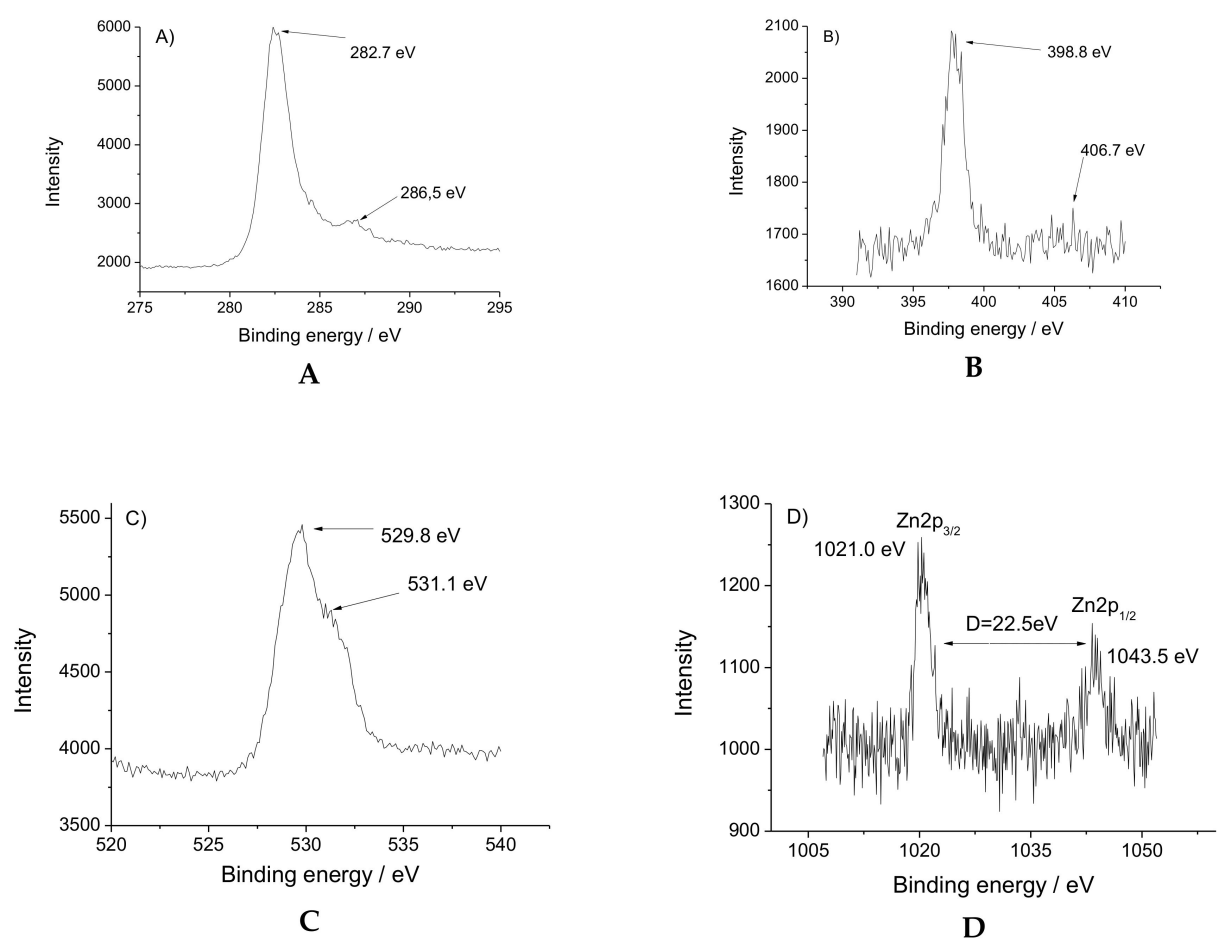
High-resolution XPS spectra of [Zn(BT)(NO_3_)_2_]: (**A**) C1s; (**B**) N1s; (**C**) O1p; (**D**) Zn2p.

The high-resolution XPS spectra of nitrogen, oxygen, carbon and zinc atoms are presented in Figure 3. The Zn(II) ions have two peaks with binding energy at 1021.0 and1043.5 eV for 2p_3/2_ and 2p_1/2_ spin orbital photoelectrons (Figure 3A). The asymmetric peak of O1s is shown in Figure 3B. The peak at 529.8 eV is considered as a C–O bond, while the binding energy at 531.1 eV is attributed to the Zn–O ligation. N1s peak at 398.9 eV is assigned to NHCO from the tripod ligand and the low energy peak of 406.7 eV is referred to the N–O bond from the NO_3_ (Figure 3C). The C1s spin-orbital photoelectron is at 282.4 eV, whereas the peak with energy bind at 286.5 eV is due to the O−C=O bond (Figure 3D).

### 3.4. Colour Characterization of the Cotton Fabrics Treated with BT and [Zn(BT)(NO_3_)_2_] 

To obtain antibacterial textiles, BT and [Zn(BT)(NO_3_)_2_] were loaded onto a textile material of 100% cotton. In organic solvents both compounds have yellow colour and emit intensive yellow-greenish fluorescence emissions. This yellow colour is retained after their deposition onto the cotton fabrics at concentration 0.5 weight %. Through the DataColor System, the colour characteristics of both textile materials have been examined and the results are summarised in Table 1.

The colour in CIELab space has been determinate using three types of coordinates: (a* and b*), (x and y) or chromaticity coordinates (XYZ). The data show that the colour changes from white for the untreated cotton fabric to yellow after its treatment with BT and [Zn(BT)(NO_3_)_2_] (Table 1). Also, the colour of the cotton fabric treated with BT is more intense than that of the respective ones treated with the Zn(II) complex. In both cases, the brightness of the samples is weaker, and this effect is more pronounced in the case of the cotton fabric treated with BT. Ligand BT and its Zn(II) complex have not affinity to the cotton fabric and their retention on the surface is due to the formation of hydrogen bonds. The chemical structure of BT and [Zn(BT)(NO_3_)_2_] (Scheme 1) indicates the possible formation of such bonds between the carbonyl groups (C=O) from the tripods and cotton hydroxyl groups. In the case of the free ligand BT which contains more free carbonyl groups in its structure, the observed fictions are better than those to the [Zn(BT)(NO_3_)_2_] where the carbonyl groups participate in Zn(II) complex formation.

**Figure 4:**
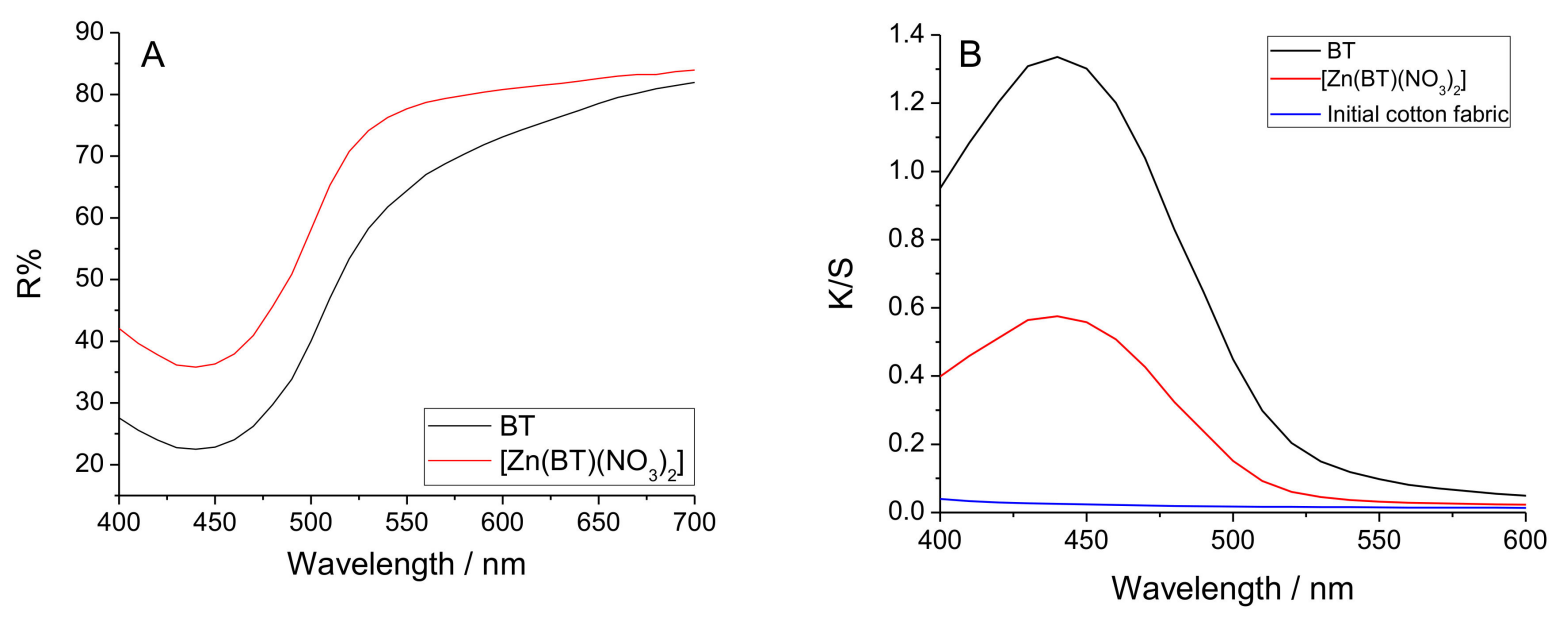
Reflectance (**A**) and K/S (ratio of the absorption coefficient (K) and the coefficient of scattering of the coloured materials (S)) (**B**) of cotton fabric treated with BT and [Zn(BT)(NO_3_)_2_].

Figure 4A presents the reflectance curves of cotton fabric treated with BT and [Zn(BT)(NO_3_)_2_]. K/S is the ratio of the absorption coefficient (K) and the coefficient of scattering of the coloured materials (S). K/S is calculated by the equation K/S = (1 − R)^2^/2R. The results obtained show that the colour of the cotton fabric treated with BT is more saturated than that of the Zn(II) complex (Figure 4B).

This result has been confirmed by measuring the colour difference (ΔE*) of the cotton fabrics treated with BT and [Zn(BT)(NO_3_)_2_]. Both compounds impart a saturated colour to the cotton fabric: BT (ΔE* = 41.23) which is preserved but to a lesser degree than in its Zn(II) complex (ΔE* = 34.57).

### 3.5. Antimicrobial Activity 

**Figure 5:**
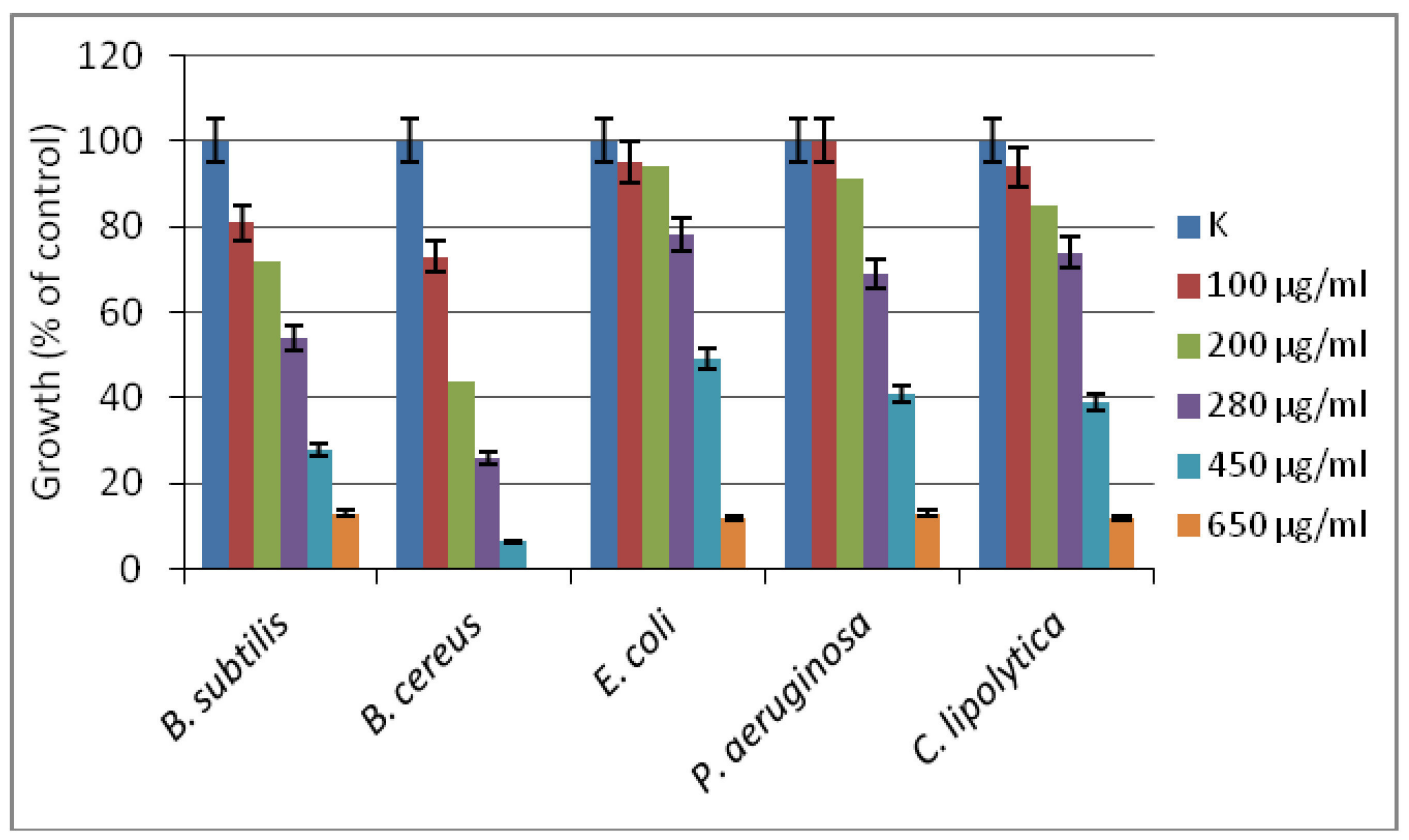
The effect of [Zn(BT)(NO_3_)_2_] on the growth of model bacteria and yeasts tested in meat-peptone broth (MPB). K, microbial growth in MPB without added compound.

The inhibitory effect of the new [Zn(BT)(NO_3_)_2_] complex has been evaluated against model yeasts and Gram-positive and Gram-negative bacterial strains. The compound has been found to be more effective against Gram-positive than against Gram-negative test cultures, with the lowest MIC_90_ of 450 µg/mL determined against *B. cereus* (Figure 5). For the other tested strains, more than 90% growth inhibition has been achieved at concentration of 650 µg/mL. According to the classification of Holetz et al. [27], the new complex [Zn(BT)(NO_3_)_2_] exhibited moderate activity against *B. cereus*, and weak activity against the other test strains. 

**Figure 6:**
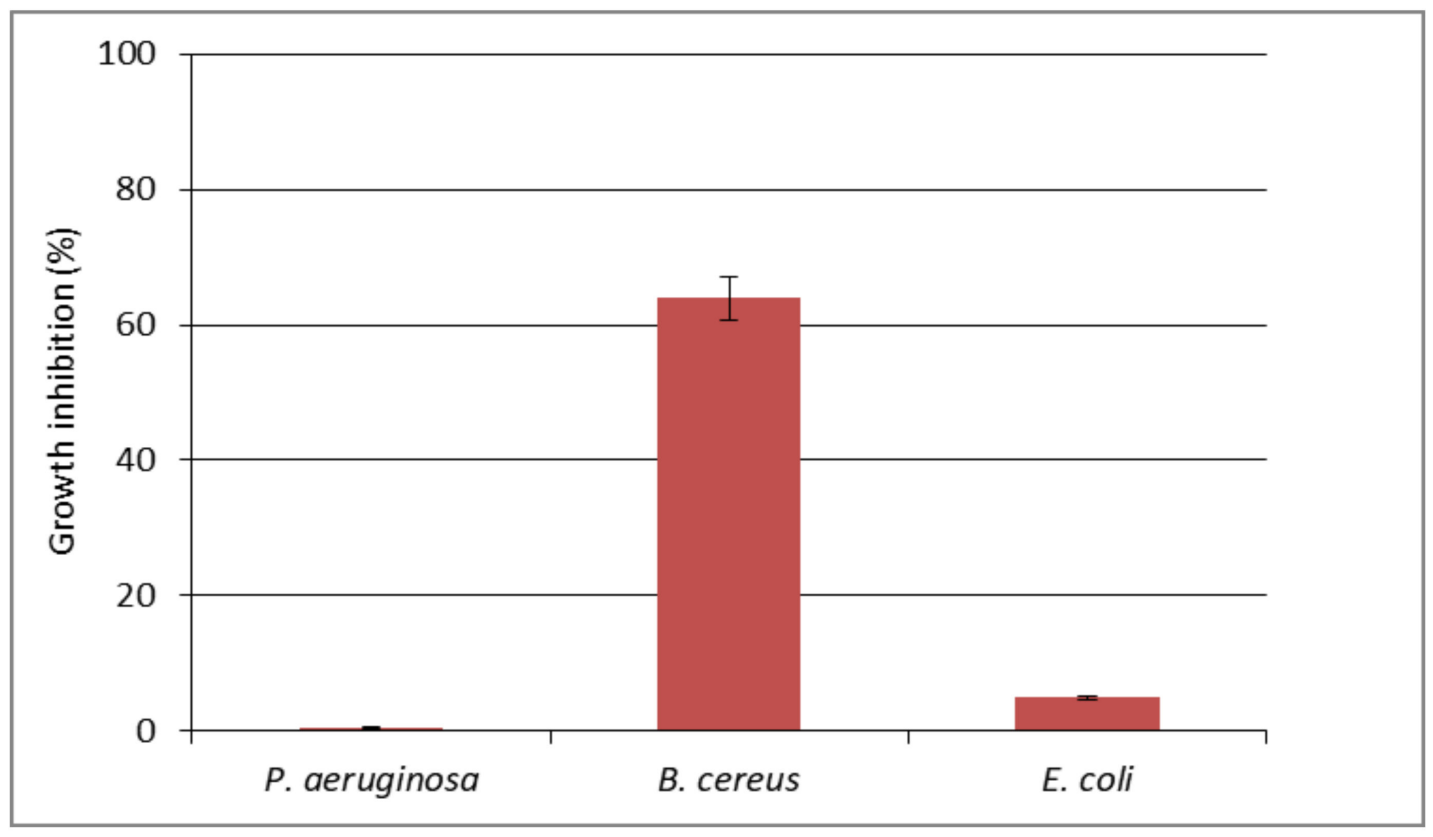
The effect of cotton textile impregnated with [Zn(BT)(NO_3_)_2_] on the growth of *P. aeruginosa, B. cereus* and *E. coli* tested in MPB.

The cotton fabric impregnated with [Zn(BT)(NO_3_)_2_] complex has been investigated for antimicrobial activity against bacterial strains *B. cereus*, *P. aeruginosa* and *E. coli* (Figure 6). The results demonstrated good antimicrobial effect of the obtained cotton-based material against *B. cereus* (about 64% growth reduction). The treated cotton sample caused only about 5% reduction of the growth of *E. coli*, and did not inhibit the growth of *P. aeruginosa*. The antimicrobial effect should be due to the direct contact of the bacteria with the surface of the cotton fabric. Several factors may affect considerably the antimicrobial effect of textiles such as mechanical retention of microbial cells on textiles dispersion of the antimicrobial material on the textile surface; variation of the hydrophobic/hydrophilic nature of textiles [28].

**Figure 7:**
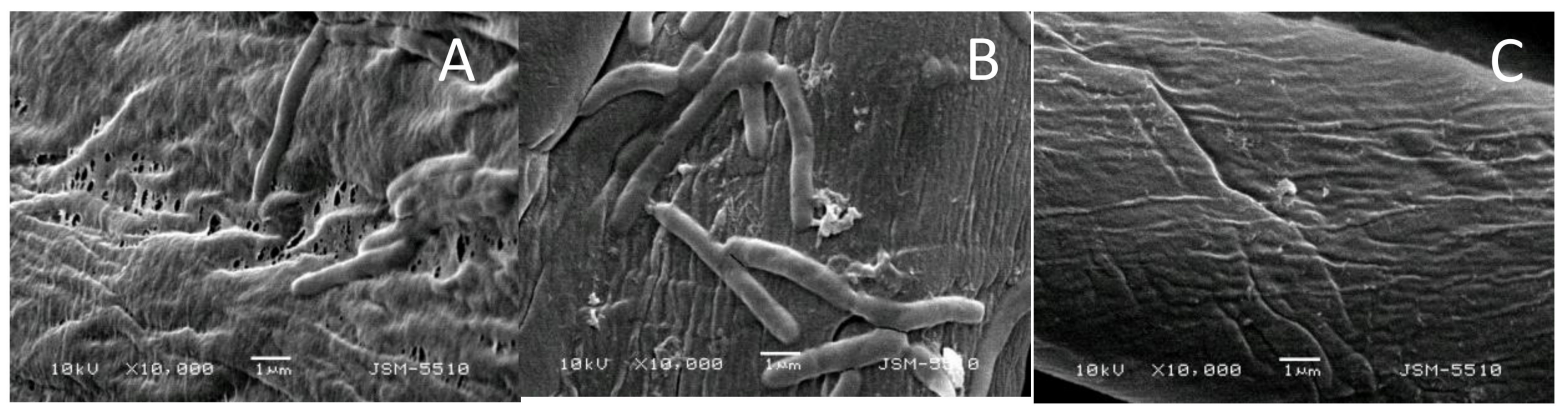
Scanning electron microscope (SEM) micrographs of cotton fabrics tested against *B. cereus*: cotton fabric before treatments (**A**), cotton fabric treated with BT (**B**), and cotton fabric treated with [Zn(BT)(NO_3_)_2_] (**C**).

The absence of ionic groups in the structure of the benzanthrone tripod makes it insoluble in water and, on the other hand, its high molecular weight does not allow it to be released easily from the cotton surface to react directly with the bacterial cells in the solution. This fact explaines low growth inhibition of the tested bacteria in solution. However, SEM observations showed that the [Zn(BT)(NO_3_)_2_] prevents the formation of bacterial biofilm onto the cotton surface (Figure 7).

The effectiveness of cotton fabrics treated with the studied tripods in preventing the adhesion and formation of bacterial biofilm was analysed by SEM. It can be seen in Figure 7A, that the untreated cotton surface is colonized by bacteria embedded in an extracellular matrix forming biofilm. Less bacterial cells and their bioproducts are observed on the textile material treated with BT (Figure 7B). The cotton fabric treated with the complex [Zn(BT)(NO_3_)_2_] is free of bacteria and no biofilm is visible (Figure 7C). These results show that [Zn(BT)(NO_3_)_2_] has very good antibacterial properties preventing the formation and proliferation of bacterial biofilm. Due to the absence of ionic groups in its structure, the complex [Zn(BT)(NO_3_)_2_] makes the cotton surface more hidrophobic. The hydrophilic character of bacteria in aqueous suspension reduces their adhesion to more hidrophobic cotton surface. This could suggest that interaction of the Zn(II) complex with the bacterial cell surface or with ions that promote microbial attachment could change cell-wall properties thus limiting microbial adhesion as a first step of biofilm formation [29].

## 4. Conclusions

A new Zn(II) complex ([Zn(BT)(NO_3_)_2_]) of a symmetrical tripod containing three benzanthrone units (BT) has been synthesized and its spectral characteristics have been investigated. The complex formed has been found to be at the 1:1 ratio of ligand to Zn (II) ions. X-ray photoelectron spectroscopy has been used for the characterisation of the chemical composition of the complex surfaces and the results confirm the presence of C, H, N, O, Zn elements in its composition. The surface morphology of BT and [Zn(BT)(NO_3_)_2_] has been studied by SEM, that has revealed the structure of the tripod to be changed significantly as a result of the formation of the zinc complex. Both compounds have been deposited onto cotton fabrics and their colour characteristics have been examined. The BT treated fabric has a more saturated colour than that of the Zn(II) complex, which is due to a change in the polarization of the benzanthrone chromophore system as a result of the complex formation. The antimicrobial activity of [Zn(BT)(NO_3_)_2_] has been investigated in vitro against various pathogenic microorganisms. It has been shown that the activity against Gram-positive bacteria is greater than against Gram-negative bacteria. The activity is preserved after deposition of compounds onto the cotton fabric. SEM analysis has shown that the Zn(II) complex prevents the formation of a bacterial biofilm over the cotton fabric which indicates its suitability for preparation of antibacterial textiles as disposable gauzes, bandages etc.

## Figures and Tables

**Table 1:** Colour characteristics of non-treated cotton fabrics and those treated with BT and [Zn(BT)(NO_3_)_2_].

-	L*	a*	b*	X	Y	Z	x	y
Cotton (control)	92.83	−0.06	2.67	78.26	82.52	84.90	0.3185	0.3361
Cotton + BT	81.97	4.14	42.35	58.81	60.24	27.20	0.4021	0.4119
Cotton + [Zn(BT)(NO_3_)_2_]	88.32	−1.75	33.12	68.16	72.74	42.39	0.3719	0.3968
